# Retinal and Circulating miRNAs in Age-Related Macular Degeneration: An *In vivo* Animal and Human Study

**DOI:** 10.3389/fphar.2017.00168

**Published:** 2017-03-30

**Authors:** Giovanni L. Romano, Chiara B. M. Platania, Filippo Drago, Salvatore Salomone, Marco Ragusa, Cristina Barbagallo, Cinzia Di Pietro, Michele Purrello, Michele Reibaldi, Teresio Avitabile, Antonio Longo, Claudio Bucolo

**Affiliations:** ^1^Department of Biomedical and Biotechnological Sciences, Section of Pharmacology, University of CataniaCatania, Italy; ^2^BioMolecular, Genome and Complex Systems BioMedicine Unit, Department of Biomedical and Biotechnological Sciences, Section of Biology and Genetics G. Sichel, University of CataniaCatania, Italy; ^3^Department of Ophthalmology, School of Medicine, University of CataniaCatania, Italy

**Keywords:** age related macular degeneration, retinal diseases, Alzheimer's disease, amyloid beta, miRNA

## Abstract

Age related macular degeneration (AMD) is the leading cause of blindness among people aged 50 and over. Retinal deposition of amyloid-β (Aβ) aggregates in AMD patients has suggested a potential link between AMD and Alzheimer's disease (AD). We have evaluated the differential retinal expression profile of miRNAs in a rat model of AMD elicited by Aβ. A serum profile of miRNAs in AMD patients has been also assessed using single TaqMan assay. Analysis of retina from rats intravitreally injected with Aβ revealed that miR-27a, miR-146a, and miR-155 were up-regulated in comparison to control rats. Seven miRNA (miR-9, miR-23a, miR-27a, miR-34a, miR-126, miR-146a, and miR-155) have been found to be dysregulated in serum of AMD patients in comparison to control group. Analysis of pathways has revealed that dysregulated miRNAs, both in the AMD animal model and in AMD patients, can target genes regulating pathways linked to neurodegeneration and inflammation, reinforcing the hypothesis that AMD is a protein misfolding disease similar to AD. In fact, miR-9, miR-23a, miR-27a, miR-34a, miR-146a, miR-155 have been found to be dysregulated both in AMD and AD. In conclusion, we suggest that miR-9, miR-23a, miR-27a, miR-34a, miR-146a, miR-155 represent potential biomarkers and new pharmacological targets for AMD.

## Introduction

Age related macular degeneration (AMD) is a common eye disease and the leading cause of blindness among people aged 50 years and older. AMD exists in dry and wet forms, the first is much more common than wet. Dry AMD is characterized by cellular debris, identified as drusen bodies, that accumulate between choroid and retina. Drusen are hallmarks of dry AMD and contain a variety of constituents, among of others, amyloid-β (Aβ) deposit. The wet form bears abnormal growth of choroidal blood vessels leading to detachment of retina along with vascular leakage and related retinal edema. AMD is listed by the World Health Organization (WHO) among the “priority eye diseases.” Besides a series of phase II/III clinical trials, up to now there is no approved treatment for dry AMD, and treatment for wet AMD is not definitive. In general, wet AMD patients experience rapid visual aberration with progression of the disease, due to vascular leakage, whereas dry AMD is often asymptomatic (Yorston, 2006; Pascolini and Mariotti, 2012). Therefore, validated microinvasive biomarkers of AMD are desirable in order to diagnose and prevent irreversible macular damage. Several single nucleotide polymorphisms (SNPs) and their association to AMD have been investigated and validated: such SNPs involve complement proteins (Gemenetzi and Lotery, 2016). Furthermore, ocular inflammatory processes have been recognized as a hallmark of AMD (Stanton and Wright, 2014; Romano et al., 2015); inflammation being a response to molecules coming from dysfunctional retinal pigment epithelium (RPE), such as lipofuscin, Aβ, carboxyethyl pyrrole. Along to these molecules found in drusen of AMD patients, C-reactive protein was found in serum of patients with severe AMD (Seddon et al., 2004, 2010; Vine et al., 2005); however, other studies have not confirmed significant increased levels of C-reactive protein in serum of AMD patients in comparison to healthy control (Dasch et al., 2005; McGwin et al., 2005). Therefore, identification and validation of serum, minimally-invasive, biomarkers of AMD are still challenging. In this perspective, differential expression of miRNAs in serum or plasma represents a potential approach to identify novel biomarkers and pharmacological targets of AMD as suggested by Berber et al. in their compelling review (Berber et al., 2017). MiRNAs are short, approximately 22-mer, non-coding RNA molecules bearing important regulatory functions, such as post-transcriptional regulation of gene expression (Bartel, 2004). Cells can secrete miRNAs that can be found stably in serum, plasma and many other biological fluids (Mitchell et al., 2008; Weber et al., 2010). Extracellular miRNAs are stable due to association to cell-derived nanovesicles (e.g., exosomes), RNA-binding proteins (e.g., Argonaute 2) or high density lipoproteins HDL (Creemers et al., 2012). Recently, miRNAs were analyzed in vitreous and plasma of exudative AMD patients, by means of non-biased miRNA arrays and validation with qPCR; miRNA-146a was found to be significantly up-regulated both in vitreous and plasma of AMD patients (Ménard et al., 2016; Berber et al., 2017). Interestingly, miRNA-146a is found also down-regulated in cerebrospinal fluid (CSF) of AD patients (Kiko et al., 2014; Müller et al., 2014; Denk et al., 2015). AMD is a complex multifactorial disease; a pathogenic mechanism common to Alzheimer's disease (AD) was postulated on the basis of Aβ deposition in drusen of AMD patients (Johnson et al., 2001; Dentchev et al., 2003; Isas et al., 2010; Romano et al., 2015; Fisichella et al., 2016). Based on this ground, several experimental models of retinal degeneration attempted to induce retinal damage and mimic AMD by using Aβ. *In vitro*, stimulation of retinal pigmented epithelium with Aβ induces expression of VEGFA (Matsui et al., 2015) and inflammatory cytokines (Liu et al., 2012, 2013; Cao et al., 2013). *In vivo*, injection of Aβ in the posterior chamber of animal eye induces inflammation (Howlett et al., 2011; Liu et al., 2013), apoptosis (Fisichella et al., 2016), and blood-retinal barrier (BRB) breakdown (Anderson et al., 2008). Because these data suggest that some common mechanism may link AMD and AD, we tested the hypothesis that the profile of miRNA expression in an animal model of Aβ-induced retinal damage and in patients affected by AMD might show some similarities. In the perspective of a translational approach, analysis of miRNAs may provide not only new insights in the pathogenic mechanisms of AMD co-shared with AD, but also novel biomarkers and pharmacological targets.

## Methods

### Literature search and selection of miRNAs to be analyzed

Extensive literature search has been carried out in order to select miRNAs (Table 1) similarly dysregulated in AMD and AD. Dysregulation of miRNAs in AMD and AD has been evaluated by accessing to miR2Disease and Human microRNA Disease Database (HMDD) and through literature search (Romano et al., 2015).

**Table 1:** **Selection of miRNAs potentially involved in AD and AMD**.

**miRNA**	**Reference AMD**	**Reference AD**
miR-9	Kutty et al., 2010; Lukiw et al., 2012	databases
miR-21	Ertekin et al., 2014	databases
miR-23a	Kutty et al., 2010; Lin et al., 2011	Lau et al., 2013; Galimberti et al., 2014
miR-24	Ertekin et al., 2014	Lugli et al., 2015
miR-27a	Wang et al., 2012	Maes et al., 2009; Sala Frigerio et al., 2013
miR-30b	Haque et al., 2012	Schonrock et al., 2010
miR-34a	Hou et al., 2013; Smit-McBride et al., 2014	databases
miR-125b	Arora et al., 2010; Lukiw et al., 2012	databases
miR-126	Bai et al., 2011	Sonntag et al., 2012
miR-146a	Lukiw et al., 2012; Kutty et al., 2013; Ménard et al., 2016; Berber et al., 2017	databases
miR-146b	Kutty et al., 2013	databases
miR-155	Saxena et al., 2015; Yan et al., 2015; Zhuang et al., 2015; Berber et al., 2017	Guedes et al., 2014
miR-210	Devlin et al., 2011; Wang et al., 2012; Szemraj et al., 2015	databases

*Databases: miR2Disease and Human microRNA Disease Database (HMDD)*.

### AMD animal model

All experiments followed the guidelines set by the Association for Research in Vision and Ophthalmology Resolution on Treatment of Animals in Research; the experimental protocol was approved by the Institutional Animal Care and Use Committee (IACUC) at University of Catania.

Human amyloid-β_1−42_ (Aβ) oligomers were freshly prepared accordingly to the following protocol:

- Reconstitution of lyophilized Aβ trifluoroacetic salt (Invitrogen, Carlsbad, CA, USA) at a concentration 1 mM in 100% 1,1,1,3,3,3-hexafluoro-2-propanol (HFIP);
- Incubation of solution at room temperature for 1 h and then sonication for 10 min in water bath sonicator;
- Removing of HIFP under gentle stream of Argon;
- Storage: vials, containing peptide Parafilm^©^-sealed at −20°C;
- Solubilization of the peptide in anhydrous dimethyl-sulfoxide (DMSO) at 5 mM final Aβ concentration;
- Dilution with sterile PBS pH 7.4, at 100 μM final Aβ concentration, and incubation for 24 h at 4°C.

This protocol is in accordance with the protocol used by several groups (Lambert et al., 2001; Dahlgren et al., 2002; Barghorn et al., 2005; Guo et al., 2007) and to the original protocol developed by Klein and co-authors (Klein et al., 2001).

Male Sprague-Dawley rats (250–300 g) were purchased from Harlan (Udine, Italy). The animals were fed with standard laboratory chow and allowed free access to water in an air controlled room with a 12-h light/12-h dark cycle. The animals were randomly divided in two experimental groups (*n* = 10): (1) control group, received intravitreal injection of 2 μl of sterile PBS pH 7.2, DMSO 2%; (2) treated group, received intravitreal injection of 0.2 nmol of Aβ (2 μl of Aβ oligomer solution). Animals were anesthetized by intravenous injection of 5 mg/kg Zoletil (2.5 mg/kg tiletamine•HCl, and 2.5 mg/kg zolazepam•HCl; Zoletil, Virbac, Milano, Italy) and 1 drop of local anesthetic (oxybuprocaine 0.4%; Novesina, Novartis, Origgio, Italy) has been administered to the eye, prior intravitreal injection (Fisichella et al., 2016). After 72 h the animals were killed and blood and retina samples collected; time slot was chosen on the basis of previously findings reporting early retinal damage 72 h after intravitreal administration of Aβ (Guo et al., 2007).

### Clinical study

The study adhered to the tenets of the Declaration of Helsinki and was approved by the Local Ethics Research Committee (*Comitato Etico Catania1*). Before starting the study, written informed consent was obtained from all patients. Eleven patients (average age 70 ± 6) were enrolled between May and July 2014 at the Retina Division of the Eye Institute of the University of Catania (Italy); active choroidal neovascularization (CNV) secondary to neovascular AMD was confirmed by fluorescein angiography. The following exclusion criteria were applied: active uveitis or ocular infection, presence of any retinopathy other than AMD, any ocular surgery within the 60 days prior to enrollment. Patients with cardiovascular disease, including a history of stroke or myocardial infarction <3 months prior to screening, uncontrolled blood pressure (defined as systolic value of >160 mmHg or diastolic value of >100 mmHg at screening at screening), diabetes mellitus, history of cancer were also excluded. For each patient with neovascular AMD, a healthy subject was enrolled as control; the control healthy subject matched the AMD patient in terms of age and sex, fulfilling the inclusion and exclusion criteria.

All enrolled subjects underwent fasting venous blood sampling. Blood samples were obtained by vein puncture using sterile and dry vacutainer tubes. Samples were centrifuged for serum isolation within 2 h from withdrawal. Whole blood was incubated for 30 min at 20°C before being centrifuged at 3,000 rpm for 15 min at 4°C. Serum was divided into aliquots and stored at −80°C until analysis.

### RNA isolation from serum and retina

Human and rat serum samples were centrifuged at 2,000 rpm for 10 min to pellet circulating cells and/or debris. MiRNAs were extracted from 400 μl serum samples by using Qiagen miRNeasy mini kit (Qiagen, Hilden, Germany), according to Qiagen supplementary protocol for purification of small RNAs from serum and plasma, and finally eluted in 40 μl of elution buffer. Total RNA, from rat retina samples, was purified by using TRIzol® reagent (ThermoFisher Scientific, Boston, MA, USA), according to the manufacturer's instructions. Quantification of RNAs was carried out by fluorometry and spectrophotometry.

### miRNA profiling by Taqman assay

We carried out RNA retro-transcription by TaqMan® MicroRNA Reverse Transcription Kit (ThermoFisher Scientific); amplification was performed by Real Time PCR with TaqMan probes (ThermoFisher Scientific), according to the manufacturer's instructions. We used miR-320a as reference gene for normalization of human serum samples (Ragusa et al., 2015), U6 and miR-16 for rat serum and retina samples (Tea et al., 2013). Real Time PCRs was carried out on a 7,900 HT Fast Real Time PCR System (Applied Biosystems, Monza, Italy). Expression fold changes were calculated by the 2^−ΔΔ*Ct*^ method (Livak and Schmittgen, 2001).

### Bioinformatic analysis of biochemical pathways

The probability of association between miRNAs and KEGG pathways was calculated through the web server DIANA-miRPath 3.0 (Romano et al., 2015), by using the miRtarbase v7 algorithm. The following groups of miRNAs were analyzed:

- miR-27a, miR-146a, miR-155
- miR-9, miR-23a, miR-27a, miR-34a, miR-126,miR-146a, miR-155
- miR-155

### Statistical analysis

GraphPad Prism (version 4.0; GraphPad Software, San Diego, CA, USA) was used for statistical analysis and graphical representation of miRNA differential expression data. Data sets were examined by one-way analysis of variance (ANOVA). Student's *T*-test was used for comparison between two groups; *p* < 0.05 were considered statistically significant.

## Results

### Dysregulation of miRNAs in AMD rat model

**Figure 1:**
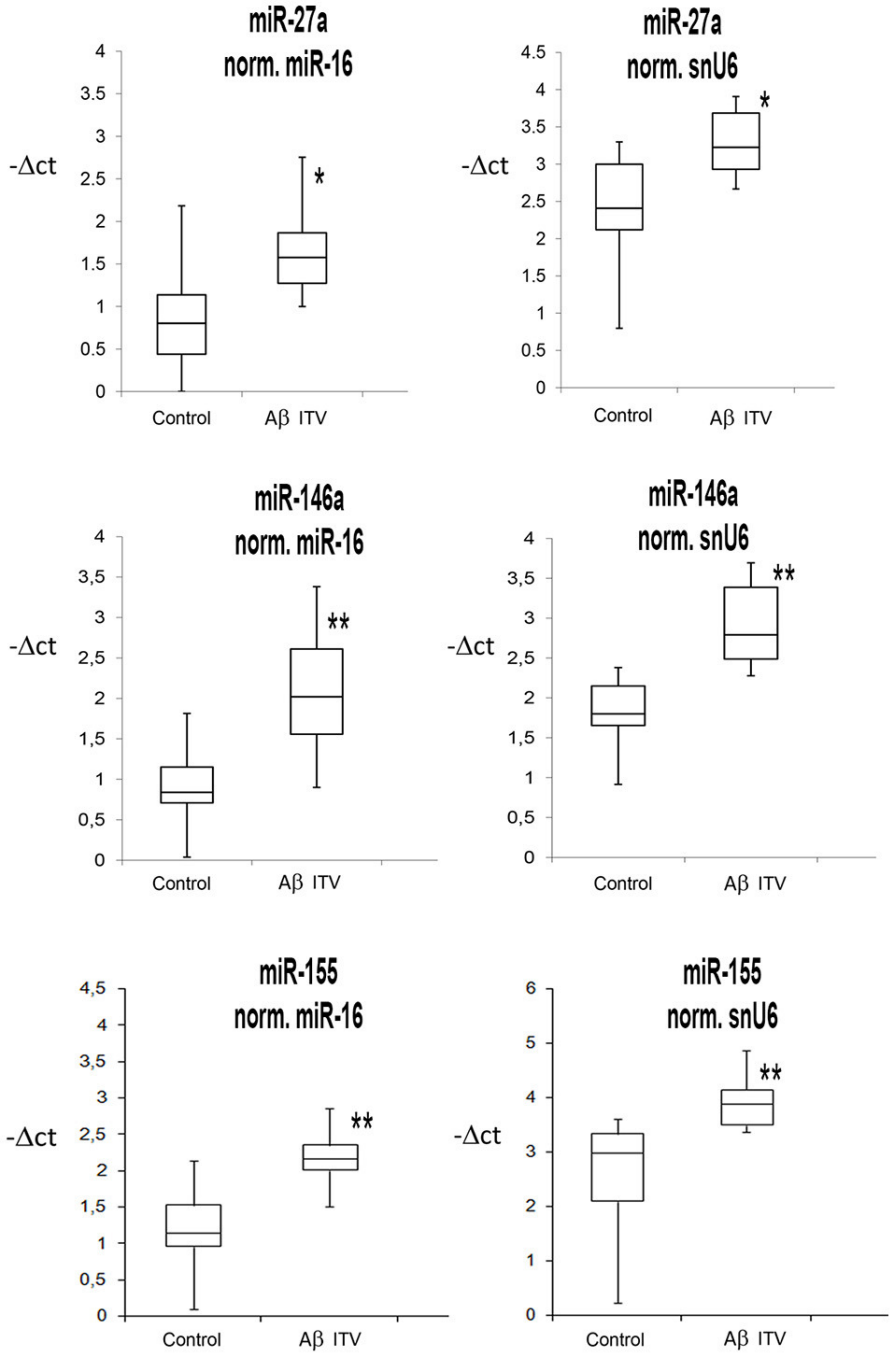
**Box Plots of miRNAs in rat retina**. Values on the y-axis are reported as –ΔCt. ^*^p < 0.05, ^**^p < 0.01 vs. control.

We analyzed the expression of 13 miRNAs (Table 1), in retina extracts from Sprague Dawley rats subjected to intravitreal injection of Aβ oligomers. This *in vivo* model of AMD has been used on the basis of previous reports showing the presence of Aβ deposition in drusen bodies of AMD patients (Seddon et al., 2004, 2010; Vine et al., 2005; Fisichella et al., 2016). Intravitreal injection of Aβ induced the up-regulation of three miRNAs in rat retina: miR-27a, miR-146a, and miR-155 (Table 2 and Figure 1). Furthermore, miR-155 was down-regulated in serum of Aβ-injected rats in comparison to controls (fold change −4.76; *p* = 0.029).

**Table 2:** **Differential expression of miRNAs, reported as fold change (FC), in retina obtained from rats injected with Aβ vs. control group**.

**miRNA**	***P*-value miR-16**	***p*-value snU6**	**FC miR-16**	**FC snU6**
miR-9	0.059	0.051		
miR-21	0.29	0.081		
miR-23a	0.041	0.067		
miR-24	0.199	0.161		
miR-27a	0.046	0.012	2.47	2.75
miR-30b	0.94	0.957		
miR-34a	0.257	0.126		
miR-125b	0.45	0.604		
miR-126	0.597	0.657		
miR-146a	0.008	0.006	2.59	2.81
miR-146b	0.096	0.154		
miR-155	0.003	0.002	3.09	3.95
miR-210	0.364	0.917		

### Dysregulation of miRNAs in patients with AMD

**Figure 2:**
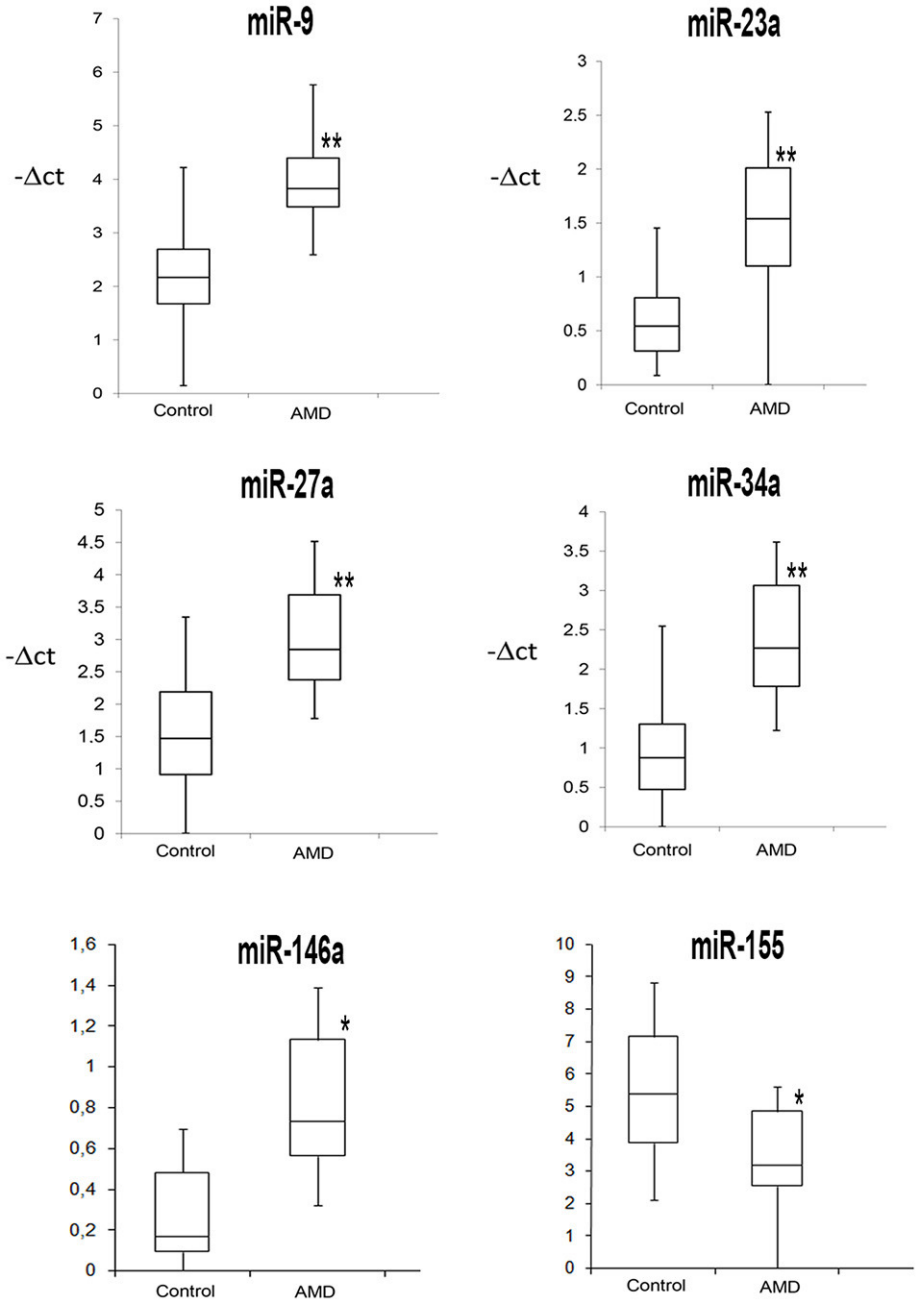
**Box Plots of miRNAs commonly dysregulated in AD and AMD patients**. Values on the y-axis are reported as −ΔCt. ^*^p < 0.05, ^**^p < 0.01 vs. control.

We analyzed the same 13 miRNA species also in serum of AMD patients (Table 3). Dysregulation of 7 miRNAs (Table 3 and Figure 2), out of 13 tested, was found in serum of AMD patients in comparison to serum of control group. In particular, up-regulation of miR-9, miR-23a, miR-27a, miR-34a, miR-126, and miR-146a was found in serum of AMD patients. On the contrary, miR-155 was down-regulated, similarly to what found in serum of rats subjected to intravitreal injection of Aβ. Table 3 shows the expression profile of miRNAs in serum of AMD patients. Figure 2 reports box-plots of six miRNAs associated to both AMD and AD.

**Table 3:** **Fold-changes (FC) of miRNA expression in AMD patients vs. control group (healthy subjects)**.

**miRNA**	***P*-value**	**FC**
miR-9	0.006	6.47
miR-21	0.065	
miR-23a	0.006	2.11
miR-24	0.06	
miR-27a	0.004	4.13
miR-30b	0.274	
miR-34a	0.007	3.28
miR-125b	0.626	
miR-126	0.019	2.97
miR-146a	0.001	1.46
miR-146b	0.124	
miR-155	0.021	−5.09
miR-210	0.070	

### Bioinformatics analysis of biochemical pathways associated to miRNAs

**Figure 3:**
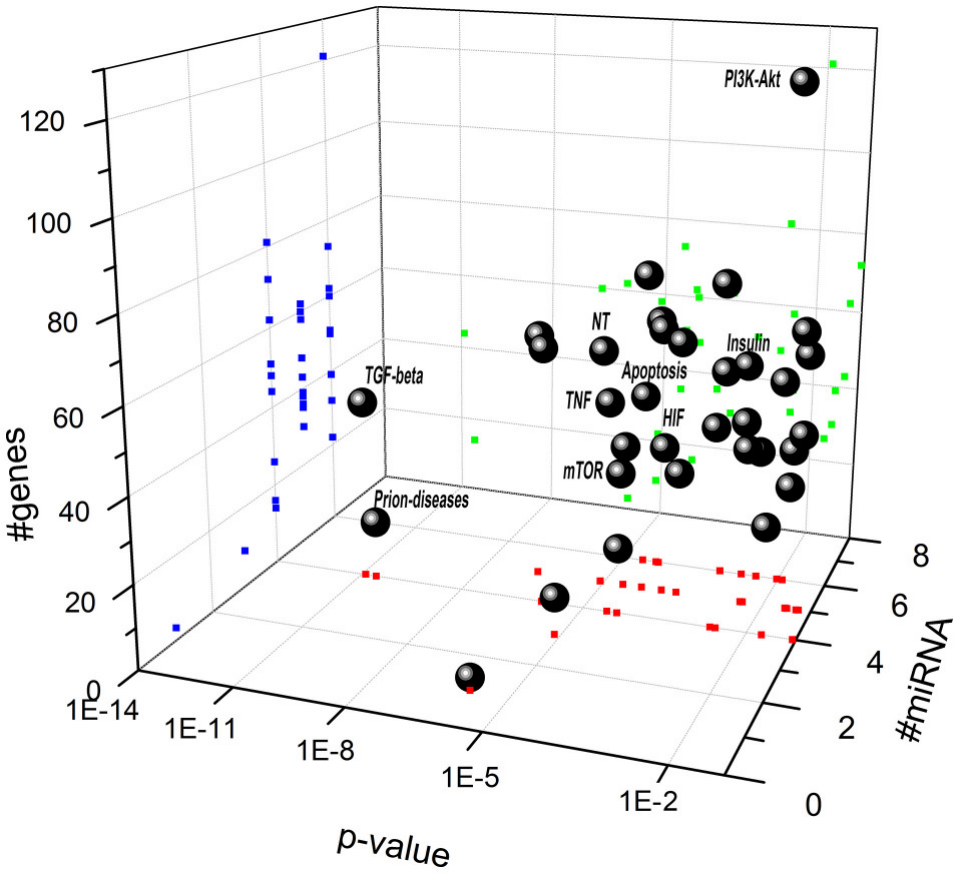
**Scatter distribution of pathways regulated by miR-27a, miR-146a, and miR-155**. Solid spheres correspond to predicted pathways. Blue (dark gray in the print version) points are the projections of # of genes, red (gray in the print version) points are the projections # of microRNA, and green (light gray in the print version) points are the projections of *p*-value associated to each pathway. (NT, neurotrophin signaling pathway; TNF, TNF signaling pathway; HIF, hypoxia inducible factor pathway).

**Figure 4:**
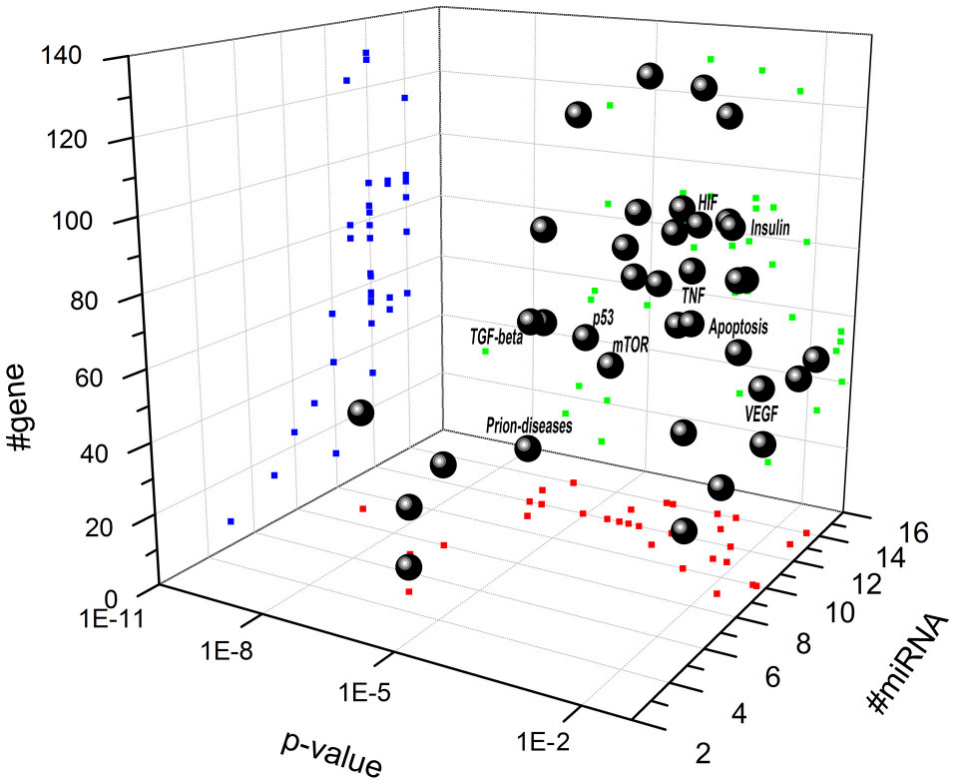
**Scatter distribution of pathways associated to miRNAs that are dysregulated in serum of AMD patients**. Solid spheres correspond to predicted pathways. Blue (dark gray in the print version) points are the projections of # of genes, red (gray in the print version) points are the projections # of microRNA, and green (light gray in the print version) points are the projections of p value associated to each pathway. (NT, neurotrophin signaling pathway; TNF, TNF signaling pathway; HIF, hypoxia inducible factor pathway; VEGF, vascular endothelial growth factor).

Biochemical pathways potentially regulated by miRNAs differentially expressed in retina of Aβ-injected rats (Figure 3 and Table 4A) and in serum of AMD patients (Figure 4 and Table 4B) have been identified through the web server DIANA-miRPath v.3. MiR-27a, miR-146a, and miR-155 (Figure 3 and Table 4A), which were up-regulated in retina of Aβ-injected rats, top scored as associated to TGF-β (*p* = 1 E-10) and prion diseases (*p* = 2 E-11) pathways. MiR-27a, miR-146a, and miR-155 have been reported to be associated to the inflammatory pathways mTOR, TNFα, HIF signaling, and NF-κB (Romano et al., 2015). Interestingly, apoptosis (*p* = 4 E-3), PI3K-AKT (*p* = 1 E-2), and p53 pathways (*p* = 2 E-2) resulted as potentially associated to the miRNAs differentially expressed in the retina of AMD animal model. These results are also consistent with our previous data (Fisichella et al., 2016) showing a deficit of TGF-β signaling in the retina of Aβ injected rats, counteracted by TGF-β1 co-administration. Furthermore, regulation of prion diseases pathway by miR-27a, miR-146a, and miR-155, reinforces the hypothesis that AMD can be a protein misfolding disease, such as AD, due to deposition of Aβ oligomers in drusen bodies. The potential link between AMD and AD is also in line with the deregulation of insulin receptor signaling by miR-27a, miR-146a, and miR-155 (Giuffrida et al., 2012; Gontier et al., 2015; Takach et al., 2015; Han et al., 2016; Sajan et al., 2016; Table 4A). The set of miRNAs differentially expressed in AMD patients can regulate the same pathways of miRNAs dysregulated in the animal model of AMD (Figure 4 and Table 4B). In fact, in AMD patients the TGF-β (*p* = 4 E-7) and prion diseases (*p* = 1 E-6) pathways were top scored, along with lipid metabolism, neurodegenerative and inflammatory pathways. Interestingly, the VEGF pathway (*p* = 3 E-2) was found to be targeted only by the set of miRNAs dysregulated in serum of AMD patients (Table 4B).

**Table 4:** **KEGG pathways target of miRNAs potentially deregulated in AMD**.

**A. miR-27a, miR-146a, miR-155**	***p*-value**	**#genes**
TGF-beta signaling pathway	1.31E-11	44
Prion diseases	2.90E-11	14
Hippo signaling pathway	2.53E-7	60
AMPK signaling pathway	1.16E-6	62
Fatty acid biosynthesis	1.50E-6	3
Fatty acid elongation	7.45E-6	9
Lysine degradation	7.73E-6	22
Neurotrophin signaling pathway	8.25E-6	58
mTOR signaling pathway	2.61E-5	35
Protein processing in endoplasmic reticulum	2.65E-5	73
TNF signaling pathway	3.20E-5	51
Fatty acid metabolism	5.17E-5	16
Signaling pathways regulating pluripotency of stem cells	5.17E-5	62
FoxO signaling pathway	5.78E-5	60
Thyroid hormone signaling pathway	6.42E-5	48
Adherens junction	1.61E-4	36
Ubiquitin mediated proteolysis	3.12E-4	62
Central carbon metabolism in cancer	3.12E-4	30
HIF-1 signaling pathway	9.92E-4	51
ErbB signaling pathway	2.38E-3	39
Sphingolipid signaling pathway	2.67E-3	48
Focal adhesion	3.26E-3	81
Insulin signaling pathway	4.31E-3	58
NF-kappa B signaling pathway	4.31E-3	32
Apoptosis	4.68E-3	38
Cell cycle	9.58E-3	50
PI3K-Akt signaling pathway	1.18E-2	121
ECM-receptor interaction	1.68E-2	26
p53signaling pathway	2.16E-2	30
Toll-like receptor signaling pathway	2.33E-2	39
Axon guidance	3.13E-2	43
RNA transport	3.38E-2	62
Endocytosis	4.53E-2	72
**B. miR-9, miR-23a, miR-27a, miR-34a, miR-126,miR-146a, miR-155**	***p*****-value**	**#genes**
Fatty acid metabolism	3.15E-1	30
Fatty acid elongation	8.96E-3	14
Fatty acid degradation	2.02E-4	24
Adherens junction	3.86E-3	53
TGF-beta signaling pathway	4.74E-4	56
Fatty acid biosynthesis	8.23E-4	7
Thyroid hormone signaling pathway	9.39E-3	84
Protein processing in endoplasmic reticulum	9.39E-3	114
Prion diseases	1.09E-5	21
p53 signaling pathway	8.77E-5	54
mTOR signaling pathway	2.91E-6	47
Hippo signaling pathway	4.40E-6	90
Neurotrophin signaling pathway	5.23E-6	82
TNF signaling pathway	7.93E-5	74
Cell cycle	1.18E-4	83
Endocytosis	1.28E-4	130
Signaling pathways regulating pluripotency of stem cells	1.58E-4	90
Sphingolipid signaling pathway	4.27E-4	76
ErbB signaling pathway	5.24E-4	62
Lysine degradation	7.50E-4	31
Axon guidance	8.49E-4	78
Estrogen signaling pathway	8.68E-4	63
Insulin signaling pathway	1.04E-3	91
Focal adhesion	1.07E-3	128
FoxO signaling pathway	1.06E-3	88
Ubiquitin mediated proteolysis	2.08E-3	89
HIF-1 signaling pathway	2.21E-3	72
Glycosaminoglycan biosynthesis - keratansulfate	2.64E-3	11
Regulation of actin cytoskeleton	4.51E-3	123
Apoptosis	5.05E-3	57
AMPK signaling pathway	7.36E-3	80
N-Glycan biosynthesis	1.41E-2	30
RNA degradation	2.41E-2	49
Fc gamma R-mediated phagocytosis	2.69E-2	55
NF-kappa B signaling pathway	2.69E-2	52
VEGF signaling pathway	3.02E-2	40
**C. miR-155**	***p*****-value**	**#genes**
TGF-beta signaling pathway	4.69E-4	11
FoxO signaling pathway	4.69E-4	26
Apoptosis	1.29E-3	20
Steroid biosynthesis	2.13E-3	4
NF-kappa B signaling pathway	2.13E-3	17
TNF signaling pathway	2.14E-3	22
Inflammatory bowel disease (IBD)	4.66E-3	13
Adherens junction	9.67E-3	14
Fatty acid elongation	1.13E-2	4
Signaling pathways regulating pluripotency of stem cells	1.19E-2	25

Analysis of serum of Aβ-injected rats revealed that one miRNA out of 13, miR-155 was down-regulated in comparison to control rats, similarly to what was found in serum of AMD patients. We have looked at pathways that can be associated to miR-155 (Table 4C). The miR-155 can regulate genes involved in the TGF-β signaling pathway (*p* = 4 E-4), in the apoptosis pathway (*p* = 1 E-3) and several inflammatory pathways, that are also regulated by the set of miRNAs differentially expressed in Aβ-injected rats and in AMD patients.

## Discussion

Thirteen miRNAs (Table 1) have been selected on the basis of previous reports on their role in AD and AMD. Analysis of these 13 miRNAs revealed that 7 miRNAs showed a significant up-regulation in serum of AMD patients in comparison to control group (miR-9, miR-23a, miR-27a, miR-34a, miR-146a, miR-155, and miR-126). Three miRNAs were found to be dysregulated both in AMD patients and in retina of Aβ-injected rats (miR-27a, miR-146a, miR-155). Incidentally, we showed that changes in circulating levels of some miRNAs (miR-9, miR-23a, miR-27a, miR-34a, miR-126, miR-146a, miR-155) as found in AMD patients are associated to Alzheimer's disease and modulate genes involved in neurodegenerative and inflammatory pathways.

In accordance to the finding of Ménard et al. (2016), we found that miR-146a is up-regulated in plasma of AMD patients and in the retina of Aβ-injected rats; furthermore this miRNA is dysregulated also in AD patients (Kiko et al., 2014; Müller et al., 2014; Denk et al., 2015; Ménard et al., 2016). To our knowledge, dysregulation of miR-27a has not been reported by other authors before, neither in experimental animal models nor in *in vitro* models of AMD. Wang et al. (2012) suggested for the first time the potential role of miR-27a in AMD. Furthermore, involvement of miR-27 in AD was well documented by other authors (Maes et al., 2009; Sala Frigerio et al., 2013). We found down-regulation of miR-155 in serum of AMD patients and Aβ injected rats; whereas we found up-regulation of miR-155 in the retina of Aβ injected rats, along with miR-27a and miR-146a. Our results are in accordance to the report by Guedes et al. (2014), showing the up-regulation of miR-155 in hippocampal and cortical brain regions of 3-Tg AD animals as well as in cultured microglia and astrocytes treated with Aβ oligomers. Because miR-155 is associated to blood brain barrier dysfunction (Lopez-Ramirez et al., 2014), the up-regulation of miR-155 in retina of rats injected with Aβ oligomers might also influence the integrity of blood retinal barrier (BRB). MiR-155 and its angiogenic target gene CCN1 were found to alter vascular and neovascular growth in mice retina (Berber et al., 2017). Increased expression of miR-155 induced formation of neovascular tufts that growth abnormally in vitreous with concomitant retinal microglial activation (Yan et al., 2015); thus, up-regulation of miR-155 in retina of Aβ injected rats might be the triggering factor of retinal inflammation and pro-angiogenic events. We found an increased expression of miR-155 in rat retina 72 h after intravitreal injection of Aβ oligomers; this result is in agreement with a previous work by Saxena et al. (2015), who found increased levels of miR-155 in retina of rats 72 h after light-induced retinal damage.

With exception of miRNA-155, down-regulated in serum of AMD patients and in serum of Aβ injected rats, six miRNAs (miR-9, miR-23a, miR-27a, miR-34a, miR-146a, miR-126) showed an up-regulation in serum of AMD patients. Lack of differentially expressed circulating miRNAs in serum of Aβ-injected rats, other than down-regulated miR-155, could be related to variables that characterize miRNA secretion such as aging, duration and type of pathology (Creemers et al., 2012; Weilner et al., 2013).

Overall, results obtained by our translational approach reinforce the hypothesis of a link between AMD and AD and further validate the retinal degenerative model induced by intravitreal injection of Aβ oligomers (Fisichella et al., 2016).

In order to study the biological role of the miRNAs dysregulated in retina of Aβ-injected rats and in serum of AMD patients, we have predicted the combinatorial effects of miRNAs in regulation of biological relevant pathways by means of DIANA-miRPath v.3. Given miRNAs as input, Diana-miRPath gives as output the KEGG (Kyoto Encyclopedia of Genes and Genomes) pathways potentially regulated by genes targeted by input miRNAs. We found that miRNAs, dysregulated both in serum of AMD patients and retina of Aβ-injected rats, can target genes of pathways associated to neurodegenerative diseases (e.g., apoptosis, ubiquitin proteolysis, neurotrophin signaling) along with inflammatory signaling pathways (e.g., mTOR, HIF, TNFα, and VEGF signaling; Figures 3, 4). Furthermore, TGF-β signaling was one of the top scored pathways along with prion diseases pathway. Dysregulation of TGF-β pathway in AD and the protective role of TGF-β1 toward brain neuroinflammatory processes were previously reported (Caraci et al., 2008, 2011, 2015; Chen et al., 2015). Furthermore, we have previously found that TGF-β1 administration can revert the increase in Bax/Bcl2 ratio induced in rat retina following intravitreal administration of Aβ oligomers (Fisichella et al., 2016).

Thus, we postulate that Aβ retinal deposition leads to inflammatory and apoptotic events along with differential expression of miRNAs able to target genes, which, in turn, dysregulate the TGF-β pathway. In fact, miR-155 and miR-27a can target 42 genes involved in the TGF-β pathway (DIANA-miRPath), while miR-146a can target genes involved in inflammatory pathways (Toll-like receptor, NF-κB, TNF signaling pathways). Worthy of note, three KEGG pathways were more recurrent: prion diseases, TGF-beta signaling, insulin receptor signaling; such bioinformatics data reinforce the link between AMD and AD. Finally, miRNAs dysregulated both in AMD patients and in rat retina damaged by Aβ can target the TGF-β signaling pathway, leading to a putative impairment of Smad-dependent TGF-β1 signaling, in accordance with previous reports (Caraci et al., 2012a,b; Fisichella et al., 2016).

## Conclusion

In conclusion, the modified miRNA levels we found in rat retina (miR-27a, miR-146a, miR-155) and serum of AMD patients (miR-9, miR-23a, miR-34a, miR-126, miR-27a, miR-146a, miR-155) suggest that, among others, miR-27a, miR-146a, and miR-155 have an important role in AMD and could represent suitable biomarkers and appealing pharmacological targets.

## Ethics statement

Animal procedures followed guidelines of the Animal Care and Use Committee of the University of Catania, and the ARVO (Association for Research in Vision and Ophthalmology) Statement for the Use of Animals in Ophthalmic and Vision Research. This study has been conducted accordingly to the Declaration of Helsinki; informed consent was obtained from all patients after explanation of the nature and possible consequences of the study. Eleven patients (average age = 70 ± 6) were enrolled between May 2014 and July 2014 at the Retina Division of the Eye Institute of the University of Catania (Italy).

## Author contributions

Authors make substantial contributions to conception and design, and/or acquisition of data, and/or analysis and interpretation of data: CB, GR, CP, MaR, CrBa, CD, MiR, AL. Authors participate in drafting the article or revising it critically for important intellectual content: CB, GR, CP, MaR, CrBa, CD, MiR, AL, SS, FD, MP, TA. Authors give final approval of the version to be submitted and any revised version: CB, GR, CP, MaR, CrBa, CD, MiR, AL, SS, FD, MP, TA.

### Conflict of interest statement

The authors declare that the research was conducted in the absence of any commercial or financial relationships that could be construed as a potential conflict of interest.
